# Taking stock of the availability and functions of National Ethics Committees worldwide

**DOI:** 10.1186/s12910-021-00614-6

**Published:** 2021-05-10

**Authors:** Patrik Hummel, Taghreed Adam, Andreas Reis, Katherine Littler

**Affiliations:** 1 Chair of Systematic Theology II (Ethics), Friedrich-Alexander-Universität Erlangen-Nürnberg, Kochstraße 6, 91054 Erlangen, Germany; 2 Emerging Technologies, Research Prioritisation and Support Unit, Research for Health Department, World Health Organization, 20, Avenue Appia, 1211 Geneva 27, Switzerland; 3 Health Ethics and Governance Unit, Research for Health Department, World Health Organization, 20, Avenue Appia, 1211 Geneva 27, Switzerland

**Keywords:** National Ethics Committees, Research ethics, Medical ethics, Public health ethics, Health policy, Global bioethics, Deliberation

## Abstract

**Background:**

National Ethics Committees (NECs) offer important oversight and guidance functions and facilitate public debate on bioethical issues. In an increasingly globalized world where technological advances, multi-national research collaborations, and pandemics are creating ethical dilemmas that transcend national borders, coordination and the joining of efforts among NECs are key. The purpose of this study is to take stock of the current NEC landscape, their varying roles and missions, and the range of bioethical topics on which they deliberated since their inception.

**Methods:**

Data on the availability, functions, and ethical deliberations (publications) of NECs globally were gathered through a systematic search of NEC websites and through contacts known to the authors. The search was conducted in English, French, and Spanish. The data abstraction was done in Excel and included the NEC’s country, region, functions, and deliberations on bioethical issues. Deliberation topics were classified into thematic categories through an iterative process of regrouping to arrive at the main set of themes.

**Results:**

124 NECs in 100 countries were identified. 44% of the NECs are in Europe and 47% are in high-income countries. Out of the 1108 retrieved publications, 40% were on bioethics in the context of research, followed by the clinic (28%) and public health issues (22%). The top five topics of these publications were: research ethics (124; 9%), genetics and genomics (62; 6%), organ transplantation (58; 5%), assisted reproductive technology (49; 4%), and end of life (36; 3%).

**Conclusion:**

Our study makes an important contribution to understanding the current interests and functions of NECs and the range of their bioethics deliberations. By making the data publicly available through this publication, it allows users to conduct tailored analyses and queries based on their interests, and to seek and strengthen collaboration and exchange. It also makes the case for the fruitfulness of developing and maintaining a global repository of current and new deliberations to more effectively advance this field for the greater good of humanity, research, and public health.

**Supplementary Information:**

The online version contains supplementary material available at 10.1186/s12910-021-00614-6.

## Background

National Ethics Committees (NECs) provide expertise and guidance on ethical questions raised in medicine, biomedical research, and public health. The details of their missions as well as the formal, deliberative pathways through which they impact policy-making and societal debates vary [1–4]. Some report directly to the government, a ministry, or contribute to legislative processes, whereas others provide non-binding counselling without pre-defined or guaranteed authority [5]. One of the main functions of NECs is to facilitate public debate on controversial bioethical issues and to produce opinions and recommendations that can help inform the public and policy-makers. The range of NEC outputs varies from general, reflective elaborations on bioethical concepts and contexts of application, to frameworks for responsible research and innovation, to more directive, specific recommendations on the application of new biotechnologies in practice [5, 6].

In an increasingly globalized world where technological advances, multi-national research collaborations, and pandemics are creating ethical dilemmas that transcend national borders, coordination and the joining of efforts among NECs are key. An important element for better coordination and collaboration among NECs is access to their deliberations and outputs such as their statements and positions. This allows policy makers and the public to draw on them and can help to avoid inefficiencies and duplication of efforts.

At the 8th Global Summit of National Bioethics Advisory Bodies in 2010, the need to increase participation of low and middle income countries in future Global Summit meetings and to continue establishing NECs in Africa and Asia was raised as a priority [3, 7]. The Global Health Ethics Unit at the World Health Organization (WHO), which provides the permanent secretariat for the Global Summit, started developing an online database of NECs, using a self-administered online submission system accessible to NECs. The aim was to provide a free and accessible source of information on the location and published opinions of these NECs. The database was launched in 2011 and last updated in 2015 [8]. At the end of 2019, the WHO Health Ethics and Governance Unit together with the WHO Global Observatory on Health Research and Development reviewed this database and, conscious of the limitations of respondent-dependent data collection approaches and the multitude of published opinions and recommendations that are not captured by the database, decided to conduct this analysis to take stock of the current NEC landscape and the range of topics on which they deliberated since their inception. This will inform future discussions among relevant stakeholders on current capacity, knowledge, needs, and gaps to further support and advance this area.

There were two goals for this work: first, to map the availability of NECs worldwide, their range of functions, and their distribution across geographic regions and income groups; second, to explore and analyze the expertise and published viewpoints (hereafter called opinions) on bioethical topics produced by these NECs. This paper reports on the process of gathering these data and summarizes the main findings.

We expect this analysis to be of value to academics, governments, and NECs as it provides a comprehensive map of current activities and topics of interest. We also make this data available (see Additional file 1) with this publication to allow researchers, policy-makers, and the public to explore and tailor the analysis to their own needs and, more importantly, to facilitate and promote coordinated future efforts especially around guidance and deliberation processes in the area of bioethics.

## Methods

The WHO online database of NECs [8] served as a starting point for the data gathering process. It contained contact information for 112 NECs from 97 countries, and 507 opinions from 20 of these countries. The content was updated and expanded through a systematic search of the webpages of NECs, internet search engines, and contacts known to the authors using the following steps.

First, a list of NECs was compiled. Committees were categorized into the six WHO regions and country income groups, as classified by the World Bank (as of November 2019). To be included on this list, a committee had to be recognized in some way to operate on the basis of a national rather than a merely regional focus or mandate. However, it was not a necessary condition for inclusion that the committee is a governmental organization. For example, the U.K.-based Nuffield Council, a nongovernmental organization with no defined or guaranteed channels of influence [5] which does affect processes and reasonings of the U.K. government, the British public as well as the international bioethics community, was included on this list. Since this part of the study focused on the current NEC landscape, inactive or discontinued committees were included if they were operative within the past ten years, i.e., in 2010 or later.

Second, we reviewed the stated NEC missions and categorized them into one or more of the following functions:

- National Research Ethics Review Committee (protocol review): committee reviews research protocols and projects that are intended to be carried out in the country;
- National Research Ethics Committee (policy development): committee develops policies and guidelines that frame research projects in the country;
- National Bioethics Committees (no research focus): committee does not have a dedicated focus on research or specific research projects, but works on bioethical issues more generally.

Third, publications on the committee webpages were screened for inclusion into a list of opinion documents issued by NECs. To be included, documents had to articulate an opinion or guidance on a bioethical topic. As a further condition for inclusion, documents had to be available in English, French, or Spanish. A document was excluded if contents were merely descriptive and did not elaborate on a normative stance, e.g., if it contained mere descriptions of states of affairs or processes, such as the constitution of the respective committee. In contrast to the first two steps, the scope for compiling NEC opinion documents was not constrained to the current NEC landscape. Thus, publications from discontinued or inactive NECs, such as the various U.S. federal bioethics bodies [9] since the 1970s, were included as well. There was no limit on the date of publication which was as early as 1975, and the cut-off date for inclusion into this analysis was November 2019.

Retrieved documents fulfilling the inclusion criteria were tagged with the issuing NEC, country, WHO region, income group, the year of publication, and available alternative language versions. If the same document was available in more than one of the three languages stated in the inclusion criteria (English, French, Spanish), it was counted as one document.

Finally, we grouped the included documents thematically into four coarse-grained *contexts* (see Table 1): research, the clinic, public health, and other. This was followed by a more fine-grained categorization that was done in several iterations, where we assigned one main *content* per publication, resulting in a total of 98 categories. The goal was to capture the content of each document as precisely as possible while also to merge and to subsume *contents* in case of sufficient similarity or relatedness.

**Table 1:** Contexts of opinion documents (one context per document)

Context (Count)	Explanation
Research (444)	Focused on the design, implementation, and frameworks of research endeavors
Clinical contexts (311)	Focused primarily on the health of an individual
Public health (242)	Focused on societal and population-level issues, not just on particular individuals
Other (111)	Further issues and themes in bioethics or legal theory

The distribution of NECs across regions and income groups, the functions they assumed and the frequency of thematic categories on their deliberations and recommendations were then analyzed, which we describe below.

## Results

**Fig. 1:**
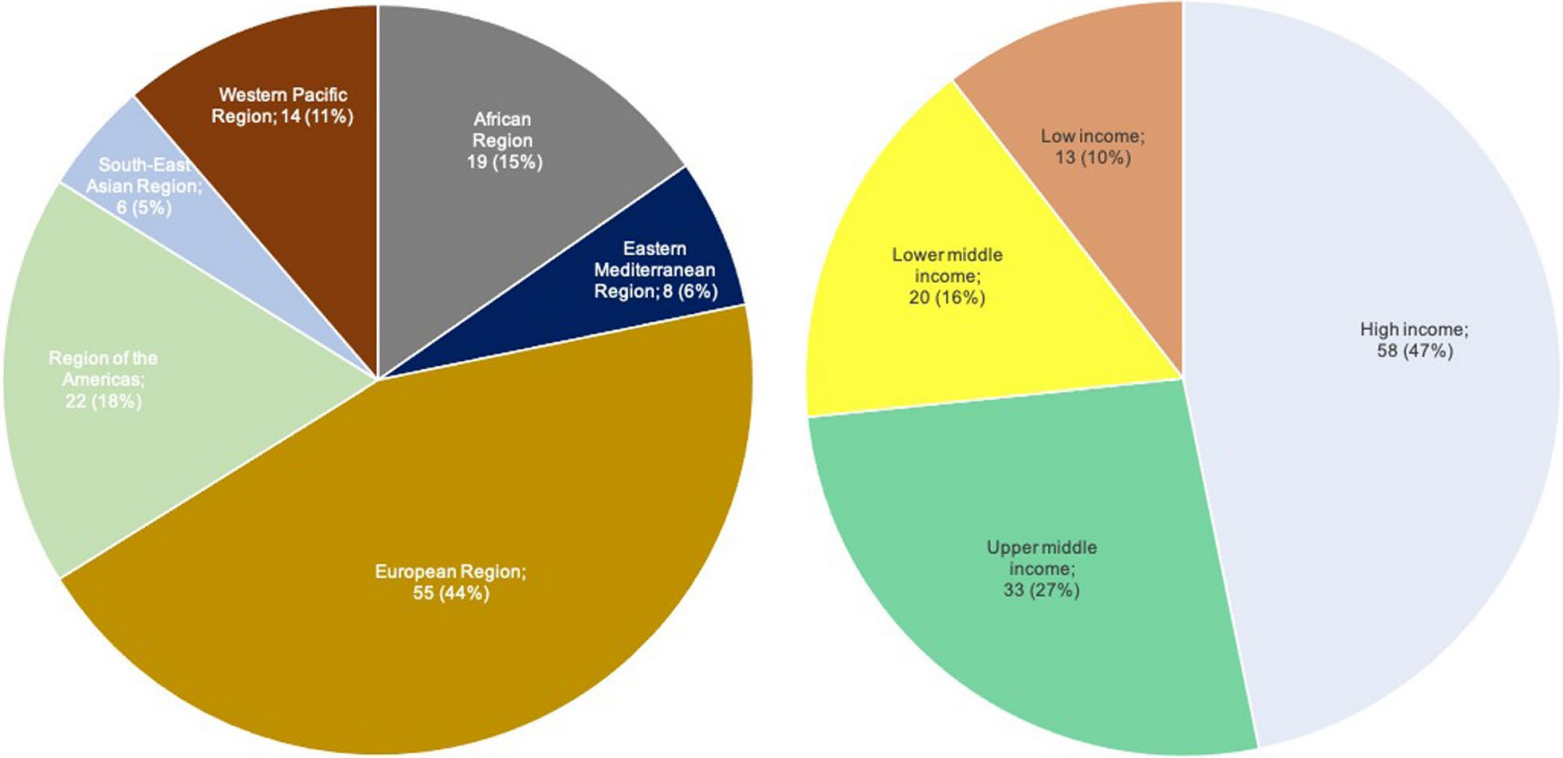
Number of Committees (n = 124) by WHO Region and Income Group

**Fig. 2:**
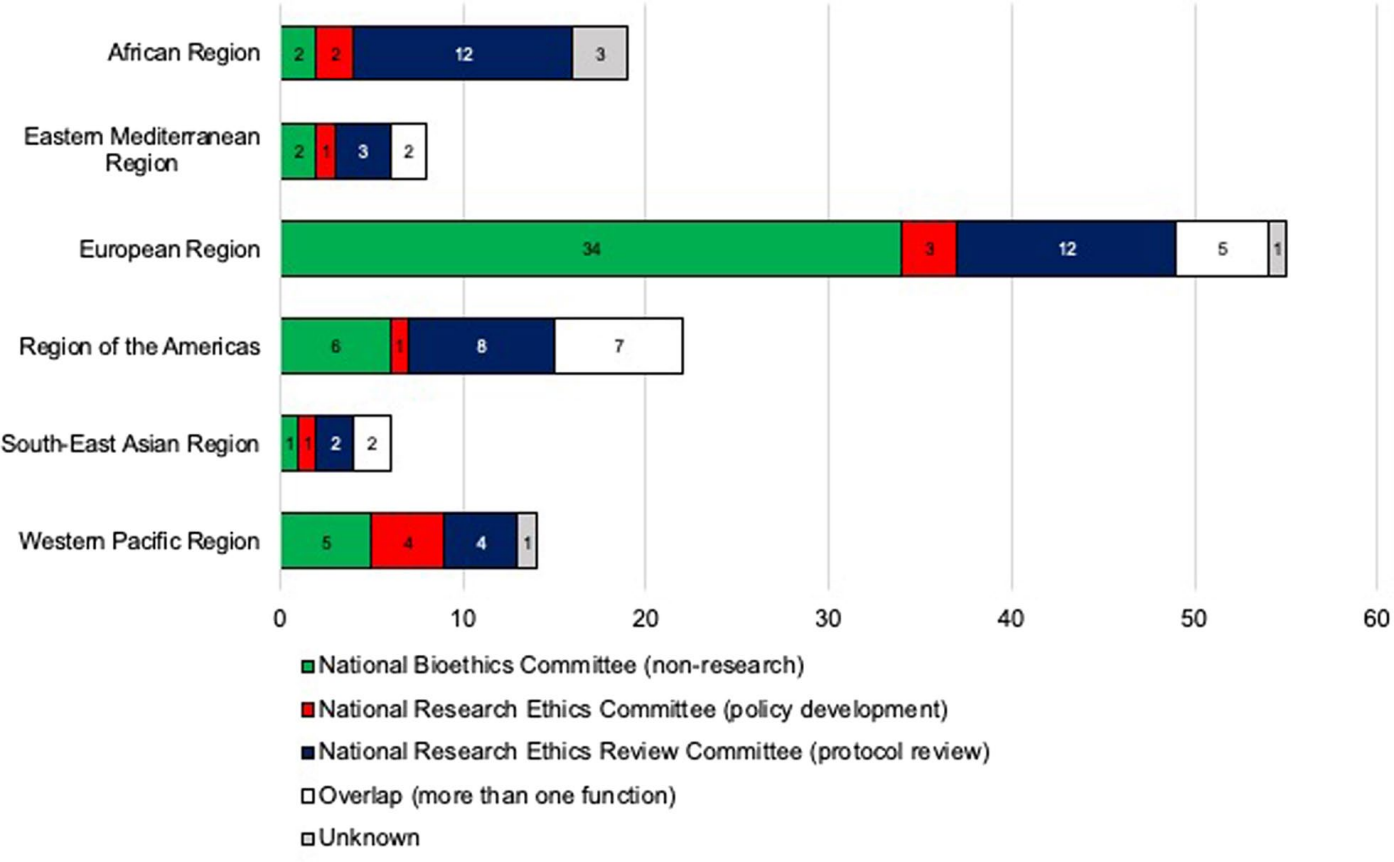
Committee functions by WHO region

The search retrieved and updated data from 100 countries and 124 committees. These NECs distribute across the European Region (44%), the Region of the Americas (18%), the African Region (15%), the Western Pacific Region (11%), the Eastern Mediterranean Region (6%), and the South-East Asian Region (5%). Most NECs are located in high-income countries (47%), followed by upper middle income (27%), lower middle income (16%), and low income countries (10%). Figure 1 illustrates the number of committees by WHO region and income group, and Fig. 2 shows the frequency of committee functions.

**Fig. 3:**
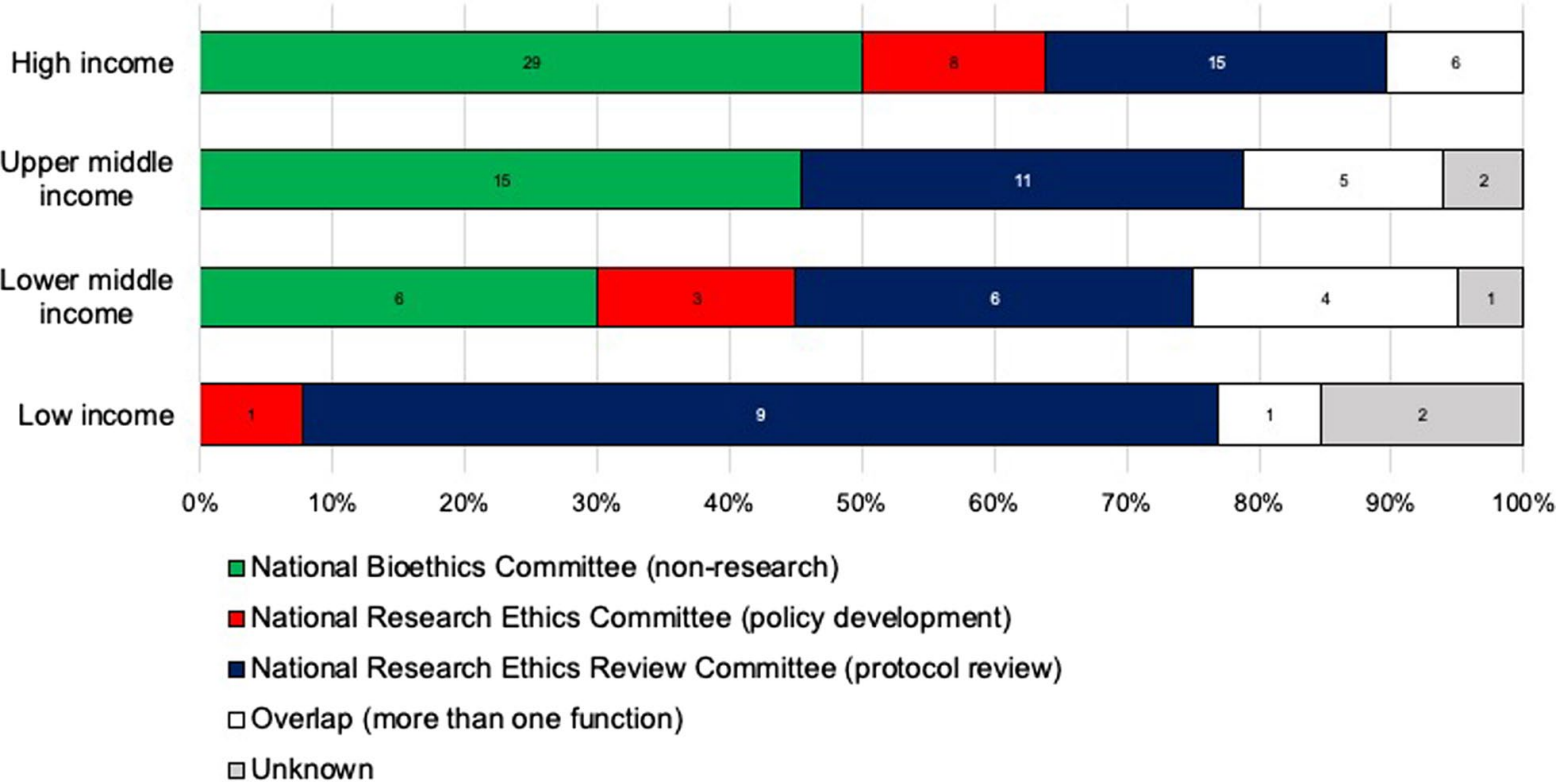
Distribution of Committee Functions by income group

With regards to the functions as defined above, 50 (40%) committees are National Bioethics Committees, 41 (33%) are National Research Ethics Review Committees that primarily review research projects and protocols, 12 (10%) are National Research Ethics Committees developing policies and guidelines that frame research projects in the respective country, and 16 (13%) fulfill more than one of these functions. The function of 5 committees was classified as ‘unknown’ since their webpages did not indicate which of the categories applies to them. As Fig. 3 illustrates, NECs from low income countries are most likely to focus on research (10 out of 13, or 77%), whereas NECs with a broader mission are more frequent in countries in the lower middle (6/20; 30%), upper middle (15/33; 45%), and high income (29/58; 50%) groups.

**Fig. 4:**
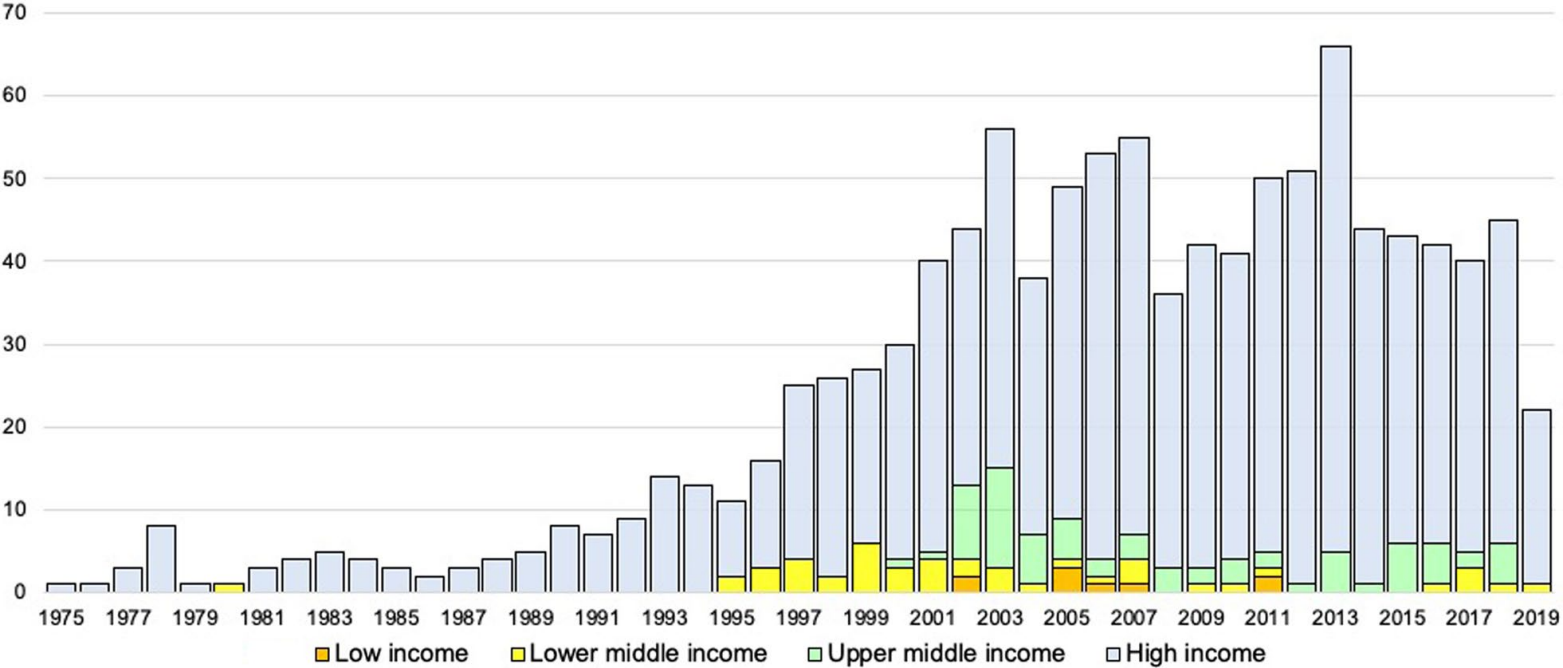
Opinion documents (n = 1108) per year, 1975–2019

The search of opinion documents retrieved 1108 relevant documents from 40 countries that were included in our analysis (some of the original 507 documents in the WHO online database of NECs [8] were replaced by newer versions or deleted if no longer publicly available). Figure 4 illustrates the growth in publication numbers of opinion documents by year and income group, which appears to be increasing steadily with more than 30 opinions per year from around the year 2000 onwards. The first document in our dataset was published by the U.S. National Commission for the Protection of Human Subjects of Biomedical and Behavioral Research in 1975. In the mid-1990s, NECs in lower-middle and upper-middle income countries started issuing opinion documents (preceded only by a 1980 statement from the Indian Council of Medical Research), followed by NECs in low-income countries around the year 2000. Overall, most of the 1108 documents are published by NECs from the European Region (810 of 1108; 73%), followed by the American Region (166; 15%), the Western Pacific Region (65; 6%), the Eastern Mediterranean Region (37; 3%) the South-East Asian Region (16; 1%), and the African Region (14; 1%).

Table 1 shows the *contexts* for which these documents were developed. Most NEC publications were concerned with bioethics in the context of research (40%), the clinic (28%), and public health (22%).

**Fig. 5:**
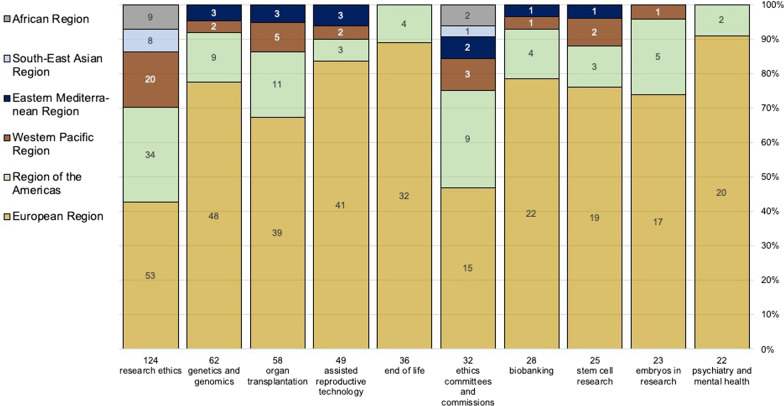
Regional Distribution of the 10 most frequent contents

Table 2 summarizes the ten most frequent *contents* for the whole period of analysis (1975–2019) and the last ten years (2009–2019). This is because almost half of the opinion pieces we retrieved were developed in the last decade. The top five most frequently debated *contents* were found to be the same for both the last decade and the whole period of analysis. These are: research ethics (124; 9%), genetics and genomics (62; 6%), organ transplantation (58; 5%), assisted reproductive technology (49; 4%), and end of life (36; 3%). New topics that emerged or increased in the last 10 years include digital health and human genome editing. Figure 5 displays the distribution of debated topics by geographical regions for the 10 most frequent *contents* published between 1975 and 2019.

**Table 2:** Most frequent contents of retrieved opinion documents (one content per document)

Most frequent *contents*, 1975–2019 (n = 1108)	Most frequent *contents*, 2009–2019 (n = 486)
1. Research ethics (124) 2. Genetics and genomics (62) 3. Organ transplantation (58) 4. Assisted reproductive technology (49) 5. End of life (36) 6. Ethics committees and commissions (32) 7. Biobanking (28) 8. Stem cell research (25) 9. Embryos in research (23) 10. Psychiatry and mental health (22)	1. Research ethics (59) 2. Organ transplantation (25) 3. Assisted reproductive technology (21) 4. End of life (18), Genetics and genomics (18) 5. Digital health (14), Biobanking (14) 6. Human genome editing (13) 7. Psychiatry and mental health (11), Public discourse and awareness (11), Ethics committees and commissions (11)

**Fig. 6:**
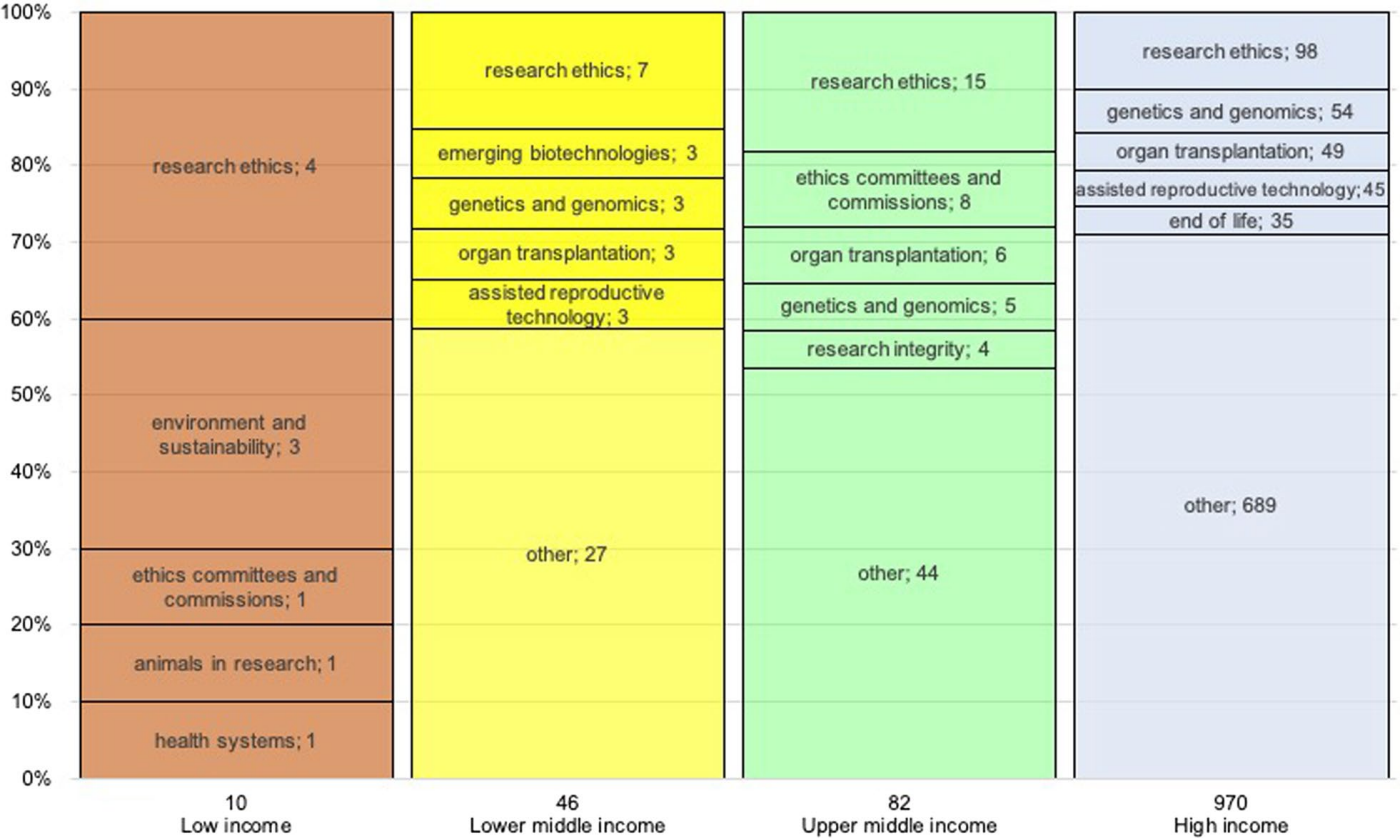
Top 5 topics per income group

Figure 6 shows the five most frequent *contents* by income group. High income countries published the most (88%; 970/1108) and addressed a relatively broad range of *contents*. The top five most frequent *contents* of interest overall are also the most relevant ones for each of the four income groups.

## Discussion

In this paper we took stock of the current landscape of National Ethics Committees (NECs) worldwide, their key functions, and what areas of bioethics they deliberated on from 1975 to the present. While some information on the constitution and activities of NECs can be found for specific countries or regions [2, 10–13], very few studies examine these on a global scale. Our study complements existing platforms such as the UNESCO Global Ethics Observatory (GEObs) [14], which contains a range of resources on global bioethics, including information on various types of ethics entities with various forms, affiliations, and functions from different countries and regions of the world, as well as a collection of legislative documents, guidelines, and codes of conduct by country. Our study by contrast focused specifically on NECs as the type of entity and on the nature of opinion documents published by them.

Our study showed that most NECs are located in the European Region (55; 44%) and high-income countries (58; 47%). It also showed a clear indication of increased capacity and interest in publishing bioethics deliberations on various topics and in various regions, with almost 50% of them published in the last decade alone. Most of these documents (88%) come from high-income countries, which might be a reflection of the duration of experience and establishment of NECs in these countries.

We share the data (see Additional file 1) as a supplemental material to allow for tailored questions to be explored by various types of users. For example, users might be interested in exploring individual NECs, their range of functions and opinions, and in getting some insights on topics of interest from individual countries. They might also want to gather and compare perspectives from different countries and regions on a specific bioethical topic, including cross-country comparisons of positions, identification of points of contention, and common and widely-shared stances. Pursuing the latter is valuable given that many timely bioethical issues, such as the example of human genome editing, apply across national boundaries and will require transnational cooperative approaches [3]. NECs provide expertise and country perspectives on these topics, reflect on conceptions held in the *status quo*, and provide recommendations on how to navigate tensions amongst the rights and interests in play. Publicity and accessibility of NEC deliberations can also help to inform public debate [15] and to facilitate inclusive consultative procedures [3, 13, 15]. Since meaningfully deliberating on potential strategies that are internationally relevant and applicable requires a common ground for conceptualizing and framing ethical issues and potential solutions, it is helpful to have access to a repository of the various deliberations on a topic as background for informed discussions. Notably, with the majority of committees (44%) and opinion documents (88%) coming from high income settings, it is important to include and to consider voices from lower resource settings when developing global positions and globally relevant guidance. In this sense, the findings of our study and the provision of the data can also help to highlight and to promote equity considerations in debates with global relevance on ethical issues in research, public health, and future advances in science and technology.

This study is not without limitations. One of the key limitations is that not all NECs were found to have functional or up-to-date webpages, which sometimes complicated completion of the committee details and functions. The amount of information and level of detail provided on NEC webpages also varies. Second, availability of opinion documents in English, French, or Spanish was a necessary condition for inclusion. The opinions of NECs that do not publish or provide translated versions in these languages are underrepresented. Third, the data counts absolute numbers of documents. It should be kept in mind that NEC publications differ in style. For example, the NECs from the United Kingdom and Germany tend to publish more extensive reports, whereas Belgium, France, the Netherlands, and others publish relatively many, more concise opinion documents. Of course, these observations are entirely neutral on productivity or the depth of published outputs, which is why caution should be taken when comparing absolute numbers of publications across different NECs.

## Conclusion

The analysis presented in this paper makes an important contribution to understanding the development of NECs over time, their current roles, interests and bioethics viewpoints. We hope that the accompanying data will facilitate tailored analyses by NECs, researchers, policy-makers, and the public to explore the state of the art of NECs, the functions they perform, and the deliberations they publish. Moreover, we hope the knowledge about current topics of interest in NEC opinions will spark new collaborations on bioethical issues that frequently transcend national boundaries and will underline the case for the utility of developing and maintaining a global repository of such information to more efficiently advance this field for the greater good of humanity, research, and public health.

## Supplementary Information

**Additional file 1:** Data set on NEC functions and published opinion documents.

## Data Availability

All data generated or analysed during this study are included in this published article and its supplementary information files.

## Abbreviations

WHO: World Health Organization
NEC: National Ethics Committee

## References

[1] Fuchs M (2005). Nationale Ethikräte. Hintergründe, Funktionen und Arbeitsweisen im Vergleich.

[2] Mali F, Pustovrh T, Groboljsek B, Coenen C (2012). National ethics advisory bodies in the emerging landscape of responsible research and innovation. NanoEthics.

[3] Gefenas E, Lukaseviciene V (2017). International capacity-building initiatives for National Bioethics Committees. Hastings Cent Rep.

[4] Köhler J, Reis AA, Saxena A (2021). A survey of national ethics and bioethics committees. Bull World Health Organ.

[5] Montgomery J (2017). The virtues of National Ethics Committees. Hastings Cent Rep.

[6] Schmidt H, Schwartz JL (2016). The missions of National Commissions: mapping the forms and functions of bioethics advisory bodies. Kennedy Inst Ethics J.

[7] Bouësseau M-C, Reis AA, Ho WC (2011). Global summit of National Ethics Committees: an essential tool for international dialogue and consensus-building. Indian J Med Ethics.

[8] World Health Organization. National Ethics Committees Database. 2011; Available from: http://apps.who.int/ethics/nationalcommittees/

[9] Capron AM (2017). Building the Next Bioethics Commission. Hastings Cent Rep.

[10] Leinhos M, The US (2005). National Bioethics Advisory Commission as a boundary organization. Sci Public Policy.

[11] Effa P, Massougbodji A, Ntoumi F, Hirsch F, Debois H, Vicari M (2007). Ethics Committees in Western and Central Africa: Concrete Foundations. Dev World Bioeth.

[12] Abou-Zeid A, Afzal M, Silverman HJ (2009). Capacity mapping of national ethics committees in the Eastern Mediterranean Region. BMC Med Ethics.

[13] Garrafa V, ten Have H (2010). National Bioethics Council: a Brazilian proposal. J Med Ethics.

[14] UNESCO. Global Ethics Observatory (GEObs). Available from: https://en.unesco.org/themes/ethics-science-and-technology/geobs

[15] Dodds S, Thomson C (2006). Bioethics and democracy: competing roles of national bioethics organisations. Bioethics.

